# Femoral Vein Diameter as a Predictor for Intravascular Volume Status

**DOI:** 10.7759/cureus.97700

**Published:** 2025-11-24

**Authors:** Kathyayini Revivarman, Nandu M, Gireesh Kumar, Arun Kumar, Sreekrishnan Trikkur, Manna M Theresa

**Affiliations:** 1 Emergency Medicine, Government Medical College, Thiruvananthapuram, Thiruvananthapuram, IND; 2 Emergency Medicine, Amrita Institute of Medical Science, Kochi, IND; 3 Department of Emergency Medicine, Dr Somervell Memorial CSI Medical College, Thiruvananthapuram, IND; 4 Emergency Medicine, Lourde Hospital, Kochi, IND

**Keywords:** central venous pressure, femoral vein diameter, fluid management, hypotensive patients, inferior vena caval diameter

## Abstract

Background

The central venous pressure (CVP) is used as a tool for assessing volume status in hypotensive patients. Ultrasonographic inferior vena cava (IVC) measurement has been accepted as a surrogate measure of CVP. But IVC visualization is occasionally cumbersome. Hence our study attempts to assess the correlation of femoral vein diameter (FVD) and CVP.

Methods

The study is a prospective observational study conducted among the hypotensive Emergency Department patients of a tertiary care centre in Kerala. Based on an earlier study sample size calculated was 27. Pregnant females, patients with deep vein thrombosis (DVT), patients with acute heart failure and patients who did not consent were excluded. IVC diameter and FVD were measured ultrasonographically. CVP was measured using a transducer. Pearson’s correlation coefficient between FVD and CVP was computed.

Results

There is a strong positive correlation between FVD and CVP (r=0.919, p<0.001). The regression equation of CVP on FVD was CVP = 0.535+9.275*FVD. FVD of 1.2cm had a sensitivity of 90% and specificity of 94.4% in predicting CVP >12mm of Hg (high CVP). FVD less than 0.8cm had a sensitivity of 25% and specificity of 100% in predicting CVP <8mm of Hg (low CVP) and had a positive predictive value of 100%.

Conclusion

The study had concluded a strong linear correlation between CVP and FVD. Hence FVD can be used as a surrogate marker of CVP to predict the volume status. FVD <0.8 cm is specific in detecting the low CVP group. FVD is of good statistical quality in predicting the high CVP group. Hence the complications of unwanted fluid resuscitation can be avoided.

## Introduction

Fluid resuscitation based on intravascular volume status forms the cornerstone of fluid management in the emergency room. There are both invasive and noninvasive methods for hemodynamic monitoring. Both inadequate and overzealous fluid correction will cause detrimental effects [1].

The central venous pressure (CVP) measurement requires invasive techniques for intravascular volume assessment. The invasive nature of the procedure and the associated complications have led to finding out new surrogates for the CVP [2]. The most common among these is inferior vena cava (IVC) diameter assessed by ultrasonography. But visualizing IVC with ultrasound is not always easy. This has led to studies for developing other alternative noninvasive tools for estimating CVP. Femoral vein diameter (FVD) measurement has hence come up as a marker for CVP. Hence, the correlation of femoral vein diameter to CVP is assessed in this study.

## Materials and methods

This is a prospective observational study done in the Emergency Medicine department of a tertiary care center in Kerala. The study was conducted after obtaining clearance from the institutional review board and ethics committee (approval IRB-AIMS-2020-010). Patients presenting to the emergency room with hypotension after confirming the inclusion and exclusion criteria were included in the study.

Hypotensive individuals (blood pressure (BP) </= 90/60 mm of Hg) with age more than 18 years who had provided written informed consent were included in the study. Pregnant females, patients diagnosed with deep vein thrombosis (DVT) of lower limb, intra-abdominal masses, acute heart failure due to cardiac/renal dysfunction confirmed by bedside echocardiogram (ECHO), urine output, and laboratory renal function tests were excluded.

Patients presenting to the emergency room with hypotension after confirming the inclusion and exclusion criteria were evaluated. The patients were evaluated as a high CVP group (CVP >12 mm of Hg) and a low CVP group (CVP <8 mm of Hg) [3]. Division of patients based on IVC collapsibility index was also done. The correlation of FVD to CVP in the two groups (IVC collapsibility index > 50% and IVC collapsibility index < 50%) was evaluated.

The sample size was estimated to detect a correlation between femoral vein diameter and central venous pressure. An expected correlation coefficient of r = 0.66, based on the findings of Cho et al., was used as the anticipated effect size [4]. The required number of subjects was determined using Fisher’s Z-transformation, which normalizes the distribution of correlation coefficients for sample size estimation. Using a 99% confidence level (α = 0.01) and 90% power (β = 0.10), the minimum required sample size was calculated to be 27 participants. 

Objectives

The primary objective of the study is to assess the correlation of CVP to femoral vein diameter. The secondary objective of the study is to assess the correlation of CVP to IVC diameter.

In patients presenting with hypotension, the initial vitals (heart rate (HR), BP) were measured from the monitor and non-invasive blood pressure (NIBP). IVC diameter, IVC collapsibility, and femoral vein diameter were measured using ultrasonography (GE LOGIQ e portable ultrasound machine; GE HealthCare, Chicago, IL, USA) by the acceptable standard technique [5,6]. Central venous pressure is measured using a transducer, according to standard technique [7].

Measurement of Inferior Vena Cava

The IVC should be identified in a transverse plane with the cardiac probe in the subxiphoid position with the probe pointing cranially. IVC is traced from its opening in the right atrium rightwards, the hepatic vein opening to IVC is sought for, and the IVC diameter is measured 2 cm to the right of this opening. The IVC collapsibility index equals (maximum IVC diameter - minimum IVC diameter)/maximum IVC diameter x 100% [8].

Measurement of Femoral Vein Diameter

By using the inguinal ligament crease, the right common femoral vein was identified with patient in dorsal decubitus position and limb in neutral rotation. After applying ultrasound gel, the great saphenous vein takeoff was traced by scanning caudally at the anterior medial aspect of the common femoral vein. When the great saphenous vein was no longer seen on ultrasonography caudally, the FVD was measured. It was measured by using the leading edge technique at the anteroposterior dimension of the common femoral vein [3,4]. To avoid the effect of probe pressure on femoral vein diameter, a standardized approach preventing significant pressure application and use of adequate amount of jelly for better visualization of femoral vein diameter is used. 

Measurement of CVP

To measure the central venous pressure, a central venous catheter is inserted into either internal jugular vein. CVP is recorded at the midaxillary line where the transducer is level with the phlebostatic axis [7].

Statistical analysis

Statistical analysis was performed using IBM SPSS version 20.0 software (IBM Corp., Armonk, NY, USA). Categorical variables were expressed using frequency and percentage. Continuous variables were summarized as mean ± standard deviation (SD) for normally distributed data or median (interquartile range) for non-normal data.

The Pearson correlation coefficient (r) was used to determine the linear relationship between FVD and CVP, and separately between IVC diameter and CVP. Correlation analysis was also performed stratified by IVC collapsibility index (>50% vs. <50%) to assess whether fluid status influenced the strength of association.

A simple linear regression model was applied to assess the predictive ability of FVD for CVP values. The regression coefficient (β), 95% confidence interval (CI), coefficient of determination (R²), and standard error of estimate were reported.

To assess the accuracy of femoral vein diameter in predicting CVP values, receiver operating characteristic (ROC) curves were plotted. Area under the curve (AUC) with 95% CI was recorded to assess discrimination. The cut-off values for femoral vein diameter corresponding to CVP more than 8 mm of Hg, 10 mm of Hg, and 12 mm of Hg were calculated from the ROC curve of FVD versus CVP using Youden's Index. Test characteristics, including sensitivity, specificity, positive predictive values, and negative predictive values, were computed. A p-value of <0.05 was considered to be statistically significant. The cut-off values of femoral vein diameter to predict the following CVP values were assessed: <8 mm of Hg, >10 mm of Hg, and >12 mm of Hg. CVP values less than 8 mm of Hg were defined as low CVP, and more than 12 mm of Hg were defined as the high CVP group [3].

## Results

Among the patients who had presented to the ER in hypotension, those who had fulfilled the inclusion criteria and after ruling out the exclusion criteria and after obtaining informed consent were enrolled into the study. Twenty-nine patients fulfilling these criteria were evaluated. Variables were measured as explained. In mechanically ventilated patients, CVP was adjusted to the positive end-expiratory pressure (PEEP).

Among the 29 patients analyzed, 62.1% (18) were males, and the remaining 37.9% were females. The mean CVP and FVD were 7.83 ± 3.39 mm of Hg and 0.79 ± 0.33 cm, respectively (Table 1).

**Table 1:** Demographic and clinical features MAP- Mean Arterial Pressure, LRTI- Lower respiratory tract infections, GI - Gastrointestinal, DKA- Diabetic ketoacidosis, CVP- central venous pressure, FVD- femoral vein diameter

Variables			
Gender		Male	18(62.1%)
		Female	11(32.9)
Age(years)			58 ± 15.77
Vitals		Heart Rate(beats/ min)	114.76 ± 17.328
		MAP(mm of Hg)	59.38 ±5.949
CVP(mm of Hg)			7.83 ± 3.39
FVD(cm)			0.79 ± 0.33
Primary diagnosis			
Sepsis			18(62.06%)
	Neutropenic sepsis		4(13.7%)
	LRTI		5(17.24%)
	GI Sepsis		2(6.89%)
	Urosepsis		4(13.7%)
	Others		3(10.34%)
DKA			2(6.89%)
GI Bleed			11(37.93%)

**Figure 1:**
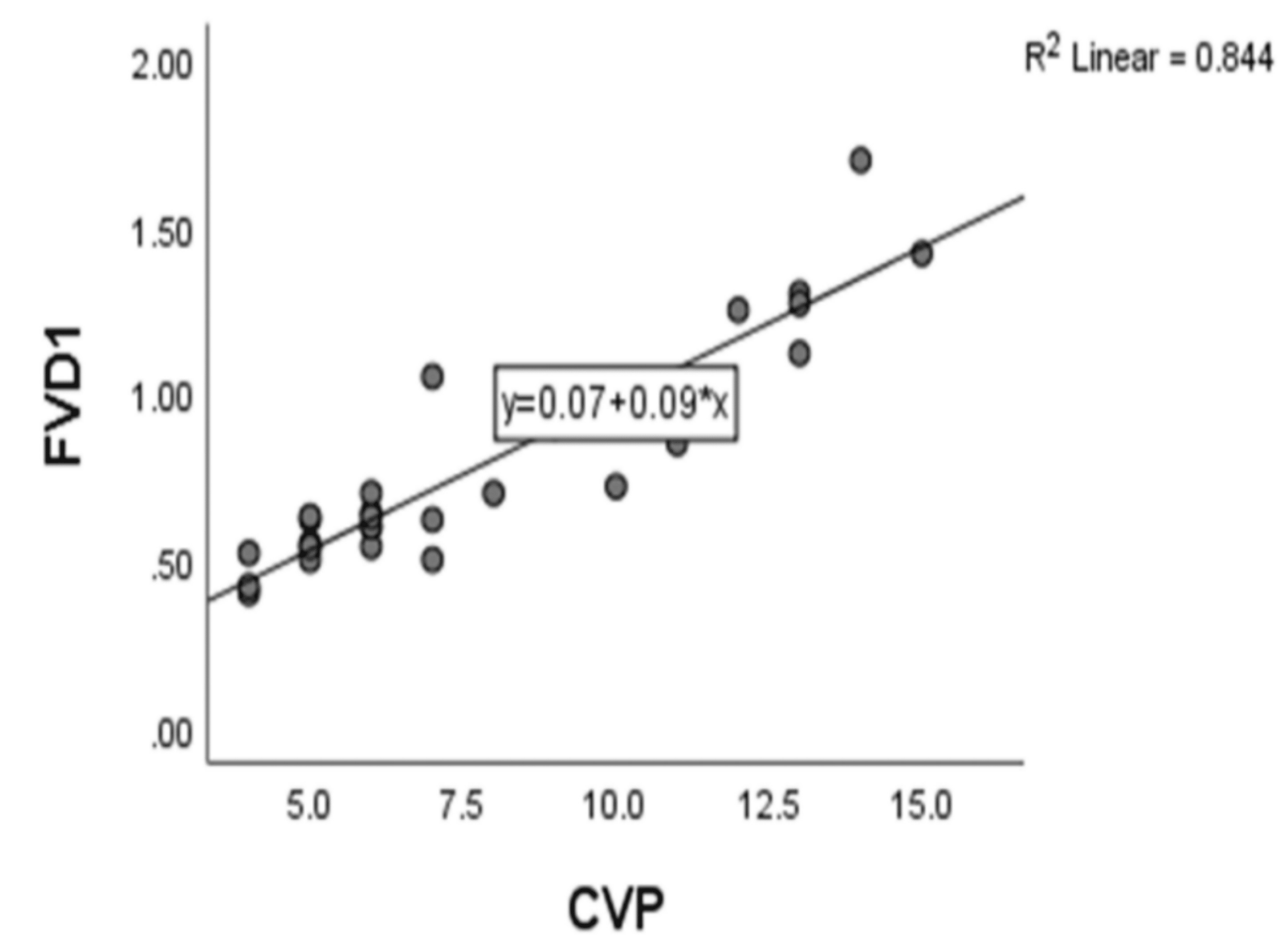
Scatter diagram between central venous pressure (CVP) and femoral vein diameter (FVD)

On correlating FVD in centimeters and CVP in mm of Hg, there is a strong positive correlation, with a correlation coefficient of r=0.919, and it was found to be statistically significant with a p value of <0.001 (Figure 1). The regression equation of central venous pressure on femoral vein diameter is CVP = 0.535 + 9.275 * FVD.

**Figure 2:**
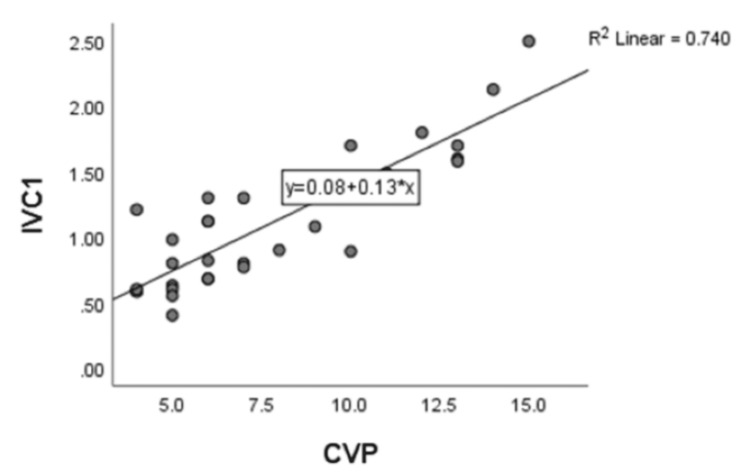
Scatter diagram between central venous pressure (CVP) and inferior vena cava (IVC)

On correlating IVC diameters in centimeters and CVP in mm of Hg, there is a strong positive correlation, with a correlation coefficient of r=0.818, and it was found to be statistically significant with a p-value of <0.001 (Figure 2).

With an area under the curve of 1.000 and a cutoff value of 1.2 cm, a femoral vein diameter of 1.2 cm had a sensitivity of 90% and a specificity of 94.4% in predicting CVP >12 mm of Hg. The accuracy was also 92.86%. With an area under the curve of 0.971 and a cutoff value of 0.8 cm, a femoral vein diameter of more than 0.8 cm had a sensitivity of 90% and a specificity of 88.89% in predicting CVP >8 mm Hg. With an area under the curve of 0.029 and a cutoff value of 0.8 cm, a femoral vein diameter less than 0.8 cm had a sensitivity of 25% and a specificity of 100% in predicting CVP <8 mm Hg. The accuracy was also only 52.63% (Table 2).

**Table 2:** Diagnostic measures for femoral vein diameter (FVD) in predicting central venous pressure (CVP) PPV – Positive Predictive value, NPV – Negative Predictive value

	Sensitivity	Specificity	PPV	NPV	Accuracy
FVD>1.2cm in CVP >12mm of Hg	90.00%	94.44%	90.00%	94.44%	92.86%
FVD>0.8cm in CVP >10mm of Hg	90.00%	94.44%	90.00%	94.44%	92.86%
FVD<0.8cm in CVP <8mm of Hg	25.00%	100.00%	100%	43.75%	52.63%

The correlation between CVP and FVD was computed in two groups, i.e., patients with IVC collapsibility more than 50% and IVC collapsibility less than 50%. On correlating FVD in centimeters and CVP in mm of Hg in the group with an IVC collapsibility index less than 50%, there is a strong positive correlation, with a correlation coefficient (r) of 0.878, and it was found to be highly statistically significant with a p-value of 0.004. Similarly, there is a strong positive correlation, with a correlation coefficient (r) of 0.716 and high statistical significance with a p-value of <0.001 in the group with an IVC collapsibility index of more than 50% (Table 3).

**Table 3:** Correlation between femoral vein diameter (FVD) and central venous pressure (CVP) in the two groups based on Inferior Vena Cava Collapsibility Index (IVCCI)

Group	Variable	n	CVP correlation (r)	p value
IVCCI <50%	FVD	8	0.878	0.004
IVCCI >50%	FVD	21	0.716	<0.001

## Discussion

Numerous studies have taken place in both the critical care and emergency medicine fields regarding the intravascular volume status, fluid responsiveness, and fluid resuscitation based on this. Many studies have reported about the utility of ultrasonography in measuring the inferior vena cava, femoral vein, and internal jugular vein as an estimate of central venous pressure. In this study we have computed the correlation between femoral vein diameter and central venous pressure. A higher degree of positive correlation between central venous pressure and femoral vein pressures, as proved in earlier studies, had instigated this study.

Among the 29 patients analyzed, 62.1% (18) were males and the remaining 37.9% were females. The mean age of the study population was 58 ± 15.77 years. The mean CVP and FVD were 7.83 ± 3.39 mm of Hg and 0.79 ± 0.33 cm, respectively. In a similar study conducted by Roy J et al., the mean age of the study population was 59 ± 15 years, and the mean CVP and femoral vein diameters were 10.8 ± 3.3 mm Hg and 0.94 ± 0.25 cm, respectively [4]. The mean CVP was 9.89 cm, and the mean femoral vein diameter was 0.92 cm in the study conducted by Malik et al. [2]. The variations in the mean CVP and femoral vein diameter may be because of the variations in the anatomical dimensions, race, and nonuniform age and sex distribution of the study population.

In our study there is a strong positive correlation between femoral vein diameter and central venous pressure (r=0.919, p<0.001). Previous studies had estimated different levels of correlation between femoral vein diameter and central venous pressure. In a study by Roy J et al., there was a moderate degree of correlation between the two parameters (r=0.66, p<0.001) [4]. In a similar study conducted by Malik et al., the central venous pressure and femoral venous diameter, which were controlled for age and sex, were determined to have a high positive correlation [2]. Dina Zidan and Ayman Baess, in their study, found that the CVP correlation with femoral vein diameter was 0.59 and the p-value was less than 0.001 [5]. The variations in correlation may have arisen because of the limited population in which the study was done.

Based on our study results, a femoral vein diameter of less than 0.8 cm had a sensitivity of 25% and a specificity of 100%, with an area under the curve of 0.029 in predicting a low CVP of less than or equal to 8 mm of Hg. But in a similar study by Roy J et al., a femoral vein diameter cutoff of 0.7 cm had a sensitivity of 95% and a specificity of 89% in predicting a low CVP of less than or equal to 8 mm of Hg. FVD >1.2 cm had a sensitivity of 90.00% and specificity of 94.4% in predicting CVP >12 mm of Hg. Similarly, in the study conducted by Roy J et al., FVD more than 1.2 cm had a specificity of 94% in predicting CVP >12 mm of Hg [4]. In our study the femoral vein diameter has a low sensitivity in predicting the low CVP group compared with the study by Roy J et al., where femoral vein diameter had high sensitivity in predicting CVP in both high and low CVP groups. The disparity might have arisen because of 1) the smaller sample in our study, 2) most patients in the group had received basic resuscitation from ERs or centers from where they were referred, and 3) differences in factors like age, sex, race, etc. among the participants in the two studies.

Patients with CVP less than 8 mm of Hg belong to a low CVP group and warrant fluid administration. Patients with CVP more than 12 mm of Hg are in the high CVP group, and fluid administration should be restricted. From our study, FVD of more than 1.2 cm has 90% sensitivity in predicting the high CVP group, i.e., we can avoid unwanted fluid administration in this group. FVD < 0.8 cm has a specificity of 100% in predicting CVP < 8 mm of Hg, i.e., the low CVP group. So according to our study, if FVD is less than 0.8 cm, it is safe to administer fluid. But the sensitivity is 25%, and hence, we may miss out on some patients who actually belong to the low CVP group while using FVD < 0.8 cm as a cutoff for predicting low CVP.

On correlating IVC diameters in centimeters and CVP in mm of Hg, there is a strong positive correlation, with a correlation coefficient of r=0.818, and it was found to be statistically significant with a p value of <0.001. A strong positive correlation (r=0.371, p<0.0005) was observed between the central venous pressure and maximum diameter of the inferior vena cava in a study conducted by A. Ilyas et al. among 100 critically ill medical ICU patients. The correlation between minimum IVC diameter, IVC collapsibility, and central venous pressure was also evaluated in the same study. The study also revealed a strong positive correlation (r = 0.572, p < 0.0005) between minimum IVC diameter and CVP and a strong linear negative correlation between central venous pressure and inferior vena caval collapsibility index (r = -0.827, p < 0.0005) [6].

Femoral vein diameter has a strong positive correlation to CVP in the two groups of patients, i.e., patients with an IVC collapsibility index less than 50% (r = 0.878, p = 0.004) and patients with an IVC collapsibility index more than 50% (r = 0.716, p < 0.001).

Hemodynamic monitoring and fluid status assessment can be done by both noninvasive and invasive methods. Physical examination and urine output monitoring were the initial tools. Pulse pressure variation, stroke volume variation, and the passive leg raising test are some of the novel techniques in assessing volume responsiveness [9]. CVP and pulmonary arterial pressure monitoring by invasive catheters are used in invasive hemodynamic monitoring. Due to its deficiencies and complications, newer noninvasive techniques have been developed for assessing volume status [4]. Point-of-care ultrasound (POCUS) has come up in the assessment of patients in shock. For the assessment of volume status, POCUS uses inferior vena cava assessment (IVC diameter, IVC collapsibility index), lung ultrasonography, and the latest in the row, FVD. Optimizing volemic status and prevention of the detrimental effects of tissue hypoperfusion as well as the deleterious effects of fluid overload occur by fluid status guided resuscitation [1]. Inadequate fluid therapy will lead to volume depletion, culminating in hypotension, shock, organ hypoperfusion, and acute kidney injury. Similarly, overaggressive fluid resuscitation ends up in volume overload, resulting in impaired oxygenation, edema, hypertension, and organ congestion [10]. The indications for fluid resuscitation include hypotension, reduced urine output, and inadequate cardiac output to maintain tissue perfusion [11].

Ultrasonography, because of its noninvasive nature, repeatability, easily attainable skill, cost reduction, absence of ionizing radiation, and rapid results, is an important tool that aids in both diagnosis and therapeutic procedures. It is inferred from the current study that there is a strong positive correlation between CVP and femoral vein diameter; femoral vein diameter can be used as a surrogate for CVP measurement. Femoral vein diameter can be used as a tool for assessing the intravascular volume status, but it cannot be used as a single tool in assessing fluid responsiveness. To predict the fluid responsiveness, further variables like femoral vein collapsibility have to be studied.

IVC measurements by ultrasonography require more expertise and skill due to the variations in inferior vena caval diameter with the phases of respiration. Inferior vena cava visualization is also difficult in conditions with poor window, post-surgical patients with dressings, gross ascites, intra-abdominal masses, gaseous distension obscuring the view, and obese individuals. Fluid status assessment by CVP is cumbersome since it is associated with invasive procedure-related complications, including infection, bleeding, arrhythmias, pneumothorax, etc. But such complications are not seen in ultrasonographic measurements of femoral vein diameter, and also the visualization is easier because of its superficial location and anatomical predictability due to the close proximity to the femoral artery, which can be palpated.

The study has put forward the correlation between FVD and CVP in both high CVP and low CVP groups and hence helped in establishing FVD as a marker of CVP. FVD assessment is an easy-to-perform, less time-consuming, noninvasive bedside technique and helps in predicting intravascular volume status of patients easily in a busy ER. The adverse outcome of unwanted fluid resuscitation can hence be avoided.

The major limitation of the study is the limited sample size. The study has been conducted in a small population in a single center in South India, and this compromises the external validation and regression equation’s use on a wide population group. The impact of factors like age, sex, preexisting conditions, intra-abdominal pressure, PEEP, and expiratory muscle usage on CVP and femoral vein diameter also needs to be evaluated. Subgroup analysis in different groups based on these factors should be done. Furthermore, in the study the measurements were done by a single observer; the interobserver variability of femoral vein diameter assessment should also be assessed.

## Conclusions

The study had concluded that there is strong linear correlation between CVP and femoral vein diameter and hence femoral vein diameter can be used as a surrogate in estimating the CVP to predict the volume status. FVD of less than 0.8cm has high specificity but low sensitivity in predicting low CVP group. FVD >1.2cm has both high sensitivity and specificity in predicting high CVP group. Hence femoral vein diameter assessment helps us in identifying specifically the high CVP group, where fluid administration is restricted. So the complications of unwanted fluid resuscitation can be avoided. But the sensitivity of femoral vein diameter in predicting low CVP is low and so fluid resuscitation is withheld in some patients who are actually in need of resuscitation. The study needs to be extended to larger population group with different race, sex, age groups, medical and surgical pre-existing conditions to validate the findings and to assess the impact of various factors on the correlation between femoral vein diameter and central venous pressure.
